# Longitudinal Changes of Mineral Concentrations in Preterm and Term Human Milk from Lactating Swiss Women

**DOI:** 10.3390/nu11081855

**Published:** 2019-08-09

**Authors:** Magalie Sabatier, Clara L. Garcia-Rodenas, Carlos A. De Castro, Peter Kastenmayer, Mario Vigo, Stéphane Dubascoux, Daniel Andrey, Marine Nicolas, Janique Richoz Payot, Valentine Bordier, Sagar K. Thakkar, Lydie Beauport, Jean-François Tolsa, Céline J. Fischer Fumeaux, Michael Affolter

**Affiliations:** 1 Nestlé Research, Société des Produits Nestlé SA, 1000 Lausanne, Switzerland; 2 Nestle Research Singapore Hub, Société des Produits Nestlé SA, 29 Quality Road, Singapore 618802, Singapore; 3 Clinic of Neonatology, Department Woman Mother Child, University Hospital of Lausanne, 1011 Lausanne, Switzerland

**Keywords:** human milk, term, preterm, calcium, phosphorus, magnesium, zinc, iron, copper, iodine, selenium, potassium, sodium

## Abstract

An adequate mineral supply to preterm infants is essential for normal growth and development. This study aimed to compare the mineral contents of human milk (HM) from healthy mothers of preterm (28–32 weeks) and full term (>37 weeks) infants. Samples were collected weekly for eight weeks for the term group (*n* = 34) and, biweekly up to 16 weeks for the preterm group (*n* = 27). Iron, zinc, selenium, copper, iodine, calcium, magnesium, phosphorus, potassium, and sodium were quantitatively analyzed by Inductively Coupled Plasma-Mass Spectrometry. The mineral contents of both HM showed parallel compositional changes over the period of lactation, with occasional significant differences when compared at the same postpartum age. However, when the comparisons were performed at an equivalent postmenstrual age, preterm HM contained less zinc and copper from week 39 to 48 (*p* < 0.002) and less selenium from week 39 to 44 (*p* < 0.002) than term HM. This translates into ranges of differences (min–max) of 53% to 78%, 30% to 72%, and 11% to 33% lower for zinc, copper, and selenium, respectively. These data provide comprehensive information on the temporal changes of ten minerals in preterm HM and may help to increase the accuracy of the mineral fortification of milk for preterm consumption.

## 1. Introduction

An estimated 15 million babies are born preterm (i.e., born before 37 weeks of gestation) worldwide every year, with the incidence ranging from 5% to 18% of births according to the area [1]. Very preterm infants (i.e., born before 32 weeks of gestation) represent a highly vulnerable population with complications that can lead to growth failure, lifetime disability including learning difficulties, visual and hearing impairments, or even death. This population requires a high level of medical care, including nutritional support, to offset the risk of nutritional deficiencies and associated adverse health outcomes. Indeed, term infants are generally born with adequate stores of minerals, the accrual of which occurs during the third trimester of pregnancy. A consequence of being born before term is a high risk of mineral shortfalls due to the limited stores, but also to the rapid postnatal growth, immature gastrointestinal tracts, high endogenous losses, and variable intakes [2]. Due to their essentiality, minerals need to be provided at a level to meet the in utero accretion rates to favor catch-up growth and to not compromise long-term neuronal and cognitive development [3]. The essential minerals or trace elements include iron, zinc, copper, iodine, selenium, calcium, phosphorus, and magnesium, but also potassium and sodium (non-exhaustive list). Among other physiological roles in humans, iron, zinc, copper, and iodine are essential for optimal brain development [4]; calcium, phosphorus, and magnesium are required for adequate bone health [5]; selenium, zinc, and iodine play a crucial role in thyroid hormone metabolism [2]; and potassium and sodium are key electrolytes involved in water fluxes and enzymatic functions [6]. Breastfeeding is the gold standard source of nutrition for term infants and is also highly recommended for preterm infants, as it is protective against several complications of preterm birth [7,8]. However, because of the risk of inadequate nutrient intakes, the fortification of mothers’ own milk or donor human milk (HM) is generally prescribed for very preterm infants to enhance the protein, energy, and micronutrients intake [7,8,9]. Fortification is often recommended to be continued until a postconceptional age of 40 weeks, but possibly until about 52 weeks if infants are discharged with a suboptimal weight for a postconceptional age [3]. Nevertheless, there is no consensus on nutrition after discharge from the neonatal intensive care unit (NICU) [10]. However, it may be assumed that to support adequate growth and development, breastfed preterm infants should have, at the minimum, a similar mineral intake to breastfed term infants of an equivalent developmental stage [11]. 

Optimal HM mineral fortification during an NICU stay and, potentially, after discharge, requires a precise understanding of HM mineral content and its potential sources of variability. Although decades of research have been allocated to HM mineral concentrations, most of the research has been performed on HM of mothers delivering at term, and for some elements, there are a limited number of reports on preterm HM. When available, comparisons between term HM and preterm HM have been performed at an equivalent postpartum age (i.e., lactation stage), but, to our knowledge, there is no evaluation of differences at the same postmenstrual age (i.e., infant developmental stage). As concentrations of several minerals and trace elements in milk change over lactation [12], the assumption was that the comparison by postmenstrual age could reveal unexplored differences between both groups of HM in the period that surrounds preterm infant discharge from NICU. Therefore, the objectives of the present study were (i) to precisely describe the concentration of zinc, copper, iodine, selenium, iron, calcium, phosphorus, magnesium, potassium, and sodium in HM from mothers of preterm (28–32 weeks of gestation) infants over a period of 16 weeks; (ii) to compare preterm HM mineral content with that of milk from mothers of full term (>37 weeks of gestation) infants at equivalent infant postpartum and postmenstrual ages.

## 2. Materials and Methods

### 2.1. Subjects

The aim of the study was to characterize the nutritional composition of preterm and term HM. The study design (monocentric, prospective cohort) and characteristics of the subjects were already reported elsewhere [13], with the results on longitudinal changes for other nutrients (i.e., macronutrients [14], proteins [13], lipids [15], and human milk oligosaccharide [16]). In brief, the study was conducted at the University Hospital in Lausanne (CHUV), Switzerland, between October 2013 and July 2014. The study included women older than 18 years of age and intending to breastfeed their infants for a minimum of 4 months. The preterm group included infants with a gestational age from 28 0/7 to 32 6/7 weeks and the full-term group referred to infants with a gestational age of 37 0/7 to 41 6/7 weeks. The exclusion criteria included any counter-indication to breastfeeding, the mother having been diagnosed with diabetes (type I or II) before pregnancy, alcohol or drugs consumption during pregnancy, and/or mothers having insufficient French language skills to follow the study guidelines.

Mothers who participated in the study were followed until postpartum week 16 for the preterm group, and week 8 for the term group, or until lactation discontinuation (whatever came first), by a dedicated research nurse who was qualified as a lactation consultant (International Board of Lactation Consultant, IBCLC).

The study protocol was established following the Declaration of Helsinki’s guidelines and was approved by the local Ethical Committee (Commission cantonale d’éthique de la recherche sur l’être humain du Canton de Vaud, Switzerland; Protocol 69/13, clinical study 11.39.NRC) on April 9, 2013. All the subjects participating in the study signed an inform consent before the enrollment. Registration of this trial was completed at ClinicalTrials.gov under NCT02052245. 

### 2.2. Data Collection

Neonatal demographic and delivery data were prospectively collected and recorded in electronic case report forms. The following infant characteristics were collected: single or multiple gestation, mode of delivery, sex, weight, length, head circumference, and gestational age at birth. Birth weight was monitored with an electronic scale accurate to the nearest 5 g, crown-heel length with a height gauge, and head circumference with a tape. An electronic scale was used to measure the maternal weight at delivery, whereas the maternal age, height, and weight before pregnancy were self-reported and completed during face-to-face interviews between mothers and the research nurse, and were verified by one of the two referent pediatricians (LB, CJFF).

### 2.3. Human Milk Sampling and Processing

A schematic representation of the HM sampling and processing has already been published previously [16]. Preterm HM samples were collected every 7 days ± 1 day during the first 8 weeks and then every 14 days ± 1 day during the following 8 weeks, which yielded a total of 12 samples during the 16 weeks. For term HM, a total of 8 samples were collected every 7 days ± 1 day for 8 weeks. 

Full HM expression from a single breast was performed between 6 am and noon (i.e., corresponding to first morning expression) using an electric breast pump (Symphony^®^, Medela, 6340 Baar, Switzerland). After full milk collection, the milk was homogenized. A maximum volume of 10 mL milk was reserved for analysis. Lower volumes were collected for the two first time points in the preterm group (i.e., max 5 mL). The remaining HM volumes were provided to the infant for feeding. Samples of HM for analysis were transferred by the mothers into 15 mL polypropylene tubes (Falcon^TM^, Fisher Scientific, Reinach, Switzerland), previously labelled with the subject number and collection information, and stored at −18 °C in the home freezer until being transferred to the hospital. Samples were temporarily kept at −80 °C at the hospital before shipment to the Nestlé Research Centre (Lausanne, Switzerland). To avoid multiple thawing/freezing cycles, HM samples were thawed once for splitting into 15 aliquots before storage at −80 °C until analysis. 

### 2.4. Mineral Quantification

Quantification of minerals was realized using Inductively Coupled Plasma Mass Spectrometry (ICP-MS, Nexion 300-D, PerkinElmer, SCIEX, Norwalk, CT, USA). For sodium, magnesium, phosphorous, potassium, calcium, iron, copper, zinc, and selenium, 0.7 mL of human breast milk was transferred into perfluoroalkoxy alkane vessels and mineralized in a MARS XPress microwave digestion system (CEM^®^ Corp., Matthews, NC, USA) using HNO_3_/H_2_O_2_. Mineralized samples were transferred to PE tubes, diluted with Milli Q water and germanium and tellurium were added as internal standards. Quantification was realized by ICP-MS using helium or CH_4_ as a collision or reaction gas. For iodine, 1 mL of human breast milk was treated with 1 mL tetramethylammonium hydroxide (25%) and 4.5 mL of Milli Q water in a drying oven at 90 °C for 3 h. Sample solutions were diluted to 15 mL and centrifuged at 1730 *g* for 15 min. Quantification was realized using ICP-MS in normal mode using germanium as the internal standard. Certified Reference Materials (CRM) were added to all analytical series to control the quality of the quantification.

### 2.5. Statistics

Statistical analyses were done using SAS (version 9.3, SAS Institute Inc., Cary, NC, USA, 2013) and R (version 3.2.1, R Foundation for Statistical Computing, Vienna, Austria). The longitudinal changes of mineral contents were compared in preterm HM and term HM at equivalent infant (1) postpartum ages and (2) postmenstrual ages. Mixed linear models were used for both comparisons to estimate the differences between the groups. The models used age (either postpartum or postmenstrual), term/preterm status, interaction between age and term/preterm status, sex of infant, and mode of delivery as fixed effects as follows: concentration = visit × term_status + child_sex + delivery + twin_type. There was only one case of mixed sex twins out of six “sets”. Since the milk samples analyses were conducted according to the mothers and not the infants, the statistical models used the mother as an individual and the sex of the infant was chosen as the sex of the first child (for twins). This entails that one mother is represented as having a male infant where indeed she has both (because of twins). Within-subject variability was accounted for by declaring the subject ID as a random effect. Contrast estimates of the model were calculated by comparing preterm and term HM groups at each time point. No imputation method was applied for missing data (both in between visits and loss to follow up) as the method used is adapted to handle incomplete data. A conventional two-sided test with a 5% error rate was used without adjusting for multiplicity. Mixed linear models were produced using the package nlme and contrast estimates were made with contrast package. R graphs were created using the package ggplot2.

## 3. Results

### 3.1. General Characteristics of the Subjects

The subjects’ characteristics were reported elsewhere [13,16] and are summarized in Table 1. In brief, 27 mothers having delivered 33 preterm infants and 34 mothers having delivered 34 term infants were included. In the respective groups, two (7.4% for preterm) and six (17.4% for term) mothers dropped out the study. In total, 473 HM samples, 257 from preterm and 216 from full-term infant mothers, were available for mineral analyses. Except for the rate of Caesarean delivery being higher in the preterm mothers than in the term ones, none of the baseline characteristics were significantly different between the two groups of mothers. Regarding the infants’ characteristics, thirty-six percent of the preterm births were twins. As expected, the weight, height, and head circumference were statistically significantly lower in the preterm group than in the term group, but not the sex distribution. 

### 3.2. Mineral Concentration and Longitudinal Change in Preterm and Term Human Milk at the Same Postpartum Age

**Figure 1:**
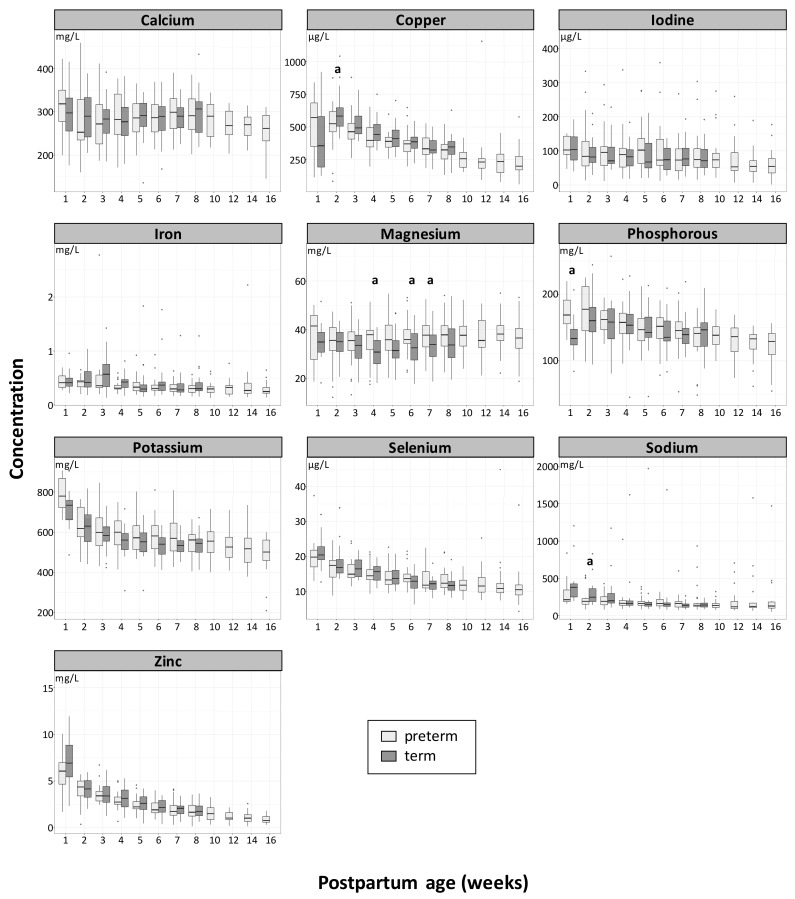
Longitudinal changes of mineral concentration (means) in term (dark grey) and preterm (light grey) human milk (HM) at an equivalent postpartum age. The letter (a) indicates if differences between term and preterm are significant *p* < 0.05. Box plots represent medians with 25th and 75th percentile, min-max range and outliers.

A summary of the results i.e., mean ± SD and median (min-max) for each mineral, is provided in Table 2 along with the range of values reported in the literature. The most concentrated element in both HMs was found to be potassium, followed by, in order of decreasing concentrations, calcium, sodium, phosphorus, magnesium, zinc, iron, copper, iodine, and selenium. The longitudinal concentrations (mean ± SD) of minerals in preterm and term HM per visit at equivalent postpartum ages are reported in Supplementary Table S1. As depicted in Figure 1, in preterm and term HM, the changes in concentration over the 4-month period of sample collection converge into three main patterns. First, the mineral concentrations were observed to increase during the first week postpartum and then to decline during the lactation, like for phosphorus and copper. Secondly, the concentrations were highest in the first week and then either decreased quickly (i.e., zinc, potassium), or gradually (i.e., calcium, selenium, sodium) over the lactation, or remained stable in mature milk (i.e., iodine, iron). Finally, for magnesium, the concentrations remained fairly stable during the course of lactation.

No statistical difference was found in mineral concentrations between preterm and term HM at the same postpartum age, except at a few time points. The phosphorus concentration was higher in preterm HM than in term HM at week 1 (*p* = 0.024). The same direction of difference was observed for magnesium at week 4 (*p* = 0.027), week 6 (*p* = 0.0357), and week 7 (*p* = 0.047). Inversely, the sodium and copper concentrations were lower in preterm HM than in term HM at week 2 (*p* = 0.032 and *p* = 0.024, respectively). The calcium to phosphorus mass ratios were found to range between 1.57 and 2.32 in preterm HM, and between 1.84 and 2.22 in term HM, with a statistically significant difference between groups found at week 1 (*p* = 0.042; preterm HM < term HM) and at week 2 (*p* = 0.018, preterm HM < term HM). The zinc to copper molar ratio ranged between 20.6 (at week 1) and 5.2 in preterm HM, and between 29.8 (at week 1) and 5.6 in term HM, with no significant difference between the two HMs. 

No significant differences were found when comparing mineral concentrations by sex, mode of delivery, or twin/single delivery. 

### 3.3. Longitudinal Changes of Mineral Concentration in Term and Preterm Human Milk at an Equivalent Postmenstrual Age

**Figure 2:**
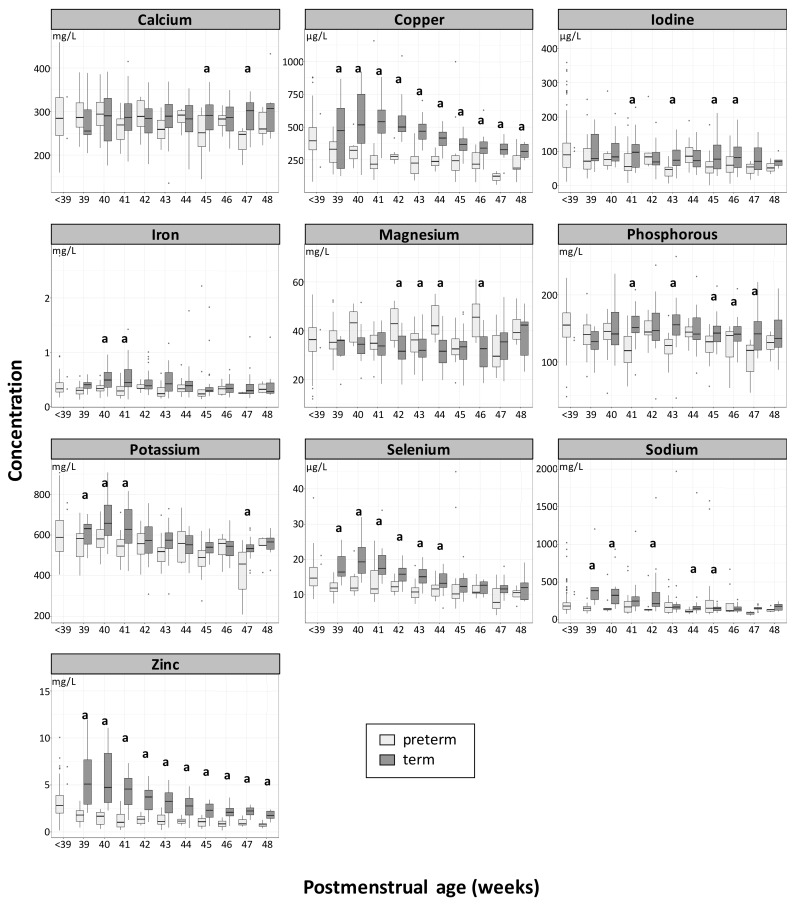
Longitudinal changes of mineral concentration (means) in term (dark grey) and preterm (light grey) human milk (HM) at an equivalent postmenstrual age. The letter (a) indicates if differences between term and preterm are significant *p* < 0.05. Box plots represent medians with 25^th^ and 75^th^ percentile, min-max range and outliers.

When the HM from mothers of preterm and full term infants was compared at an equivalent postmenstrual age, the main significant differences were observed for zinc, copper, selenium, and magnesium. As depicted in Figure 2, zinc and copper concentrations in term HM were significantly higher than in preterm HM from week 39 to 48 of postmenstrual age (*p* < 0.002). Similar observations were made for selenium, with significant differences being found at week 39 to 44 (*p* < 0.002). In comparison to term HM, preterm HM contained 53% to 78% (average 62%), 30% to 72% (average 44%), and 11% to 33% (average 24%) less zinc, copper, and selenium, respectively. 

Magnesium is the only element for which the concentration in preterm HM was found to be statistically significantly higher than in term HM from weeks 42 to 44 and week 46 (*p* < 0.05). The differences between preterm HM and term HM per postmenstrual age ranged from minus 17% to plus 39% (average + 12%). 

Statistically significant differences were also found for sodium, potassium, phosphorus, iron, iodine, and calcium at scattered postmenstrual weeks, with higher values in favor of term HM. The sodium concentration in preterm HM was significantly lower at weeks 39, 40, and 42, and weeks 44 to 45 (*p* < 0.01). The potassium concentration was significantly lower at week 39 to 41 (*p* < 0.006); for phosphorus, at week 41, 43, 45, and 47 (*p* < 0.05); for iodine, at week 41, 43, 45, and 46; for iron, at week 40 and 41 (*p* < 0.002); and for calcium, at week 45 and 47 (*p* < 0.05). The longitudinal concentrations (mean ± SD) of minerals in preterm and term HM per visit at equivalent postmenstrual ages are reported in Supplementary Table S2.

## 4. Discussion 

We have reported a comprehensive longitudinal overview of the content of 10 minerals in 500 HM samples from women delivering before term or at term. The study design enabled the comparison of preterm and term HM mineral contents at the same postpartum weeks (i.e., same lactation stages) and at equivalent postmenstrual weeks (i.e., equivalent infant developmental stages).

### 4.1. Mineral Concentration and Longitudinal Change in Preterm and Term Human Milk at the Same Postpartum Age

To our knowledge, the results of our study represent the first set of mineral analyses from breast milk in apparently healthy Swiss mothers of preterm and term infants. As illustrated in Table 2, the mean and median concentration determined for each individual element in preterm HM over the period of sample collection was found to be in the range of retrieved published values for HM [17,18,19,20,21,22,23,24,25,26,27,28]. These ranges can be wide and this variability is mainly due to the stage of lactation and to the intra and intersubject variability, including the circadian rhythm [12,29]. The presented result is in line with previous research performed in HM from a well-nourished population which showed, for several minerals, that their diet, status, or supplementation have a limited or no impact on their milk concentration. That is, for instance, the case for iron, copper, zinc, and magnesium [12], and to some extent, for phosphorus, potassium, and sodium [30,31,32]. Inversely, the calcium, iodine, and selenium concentration in HM can be impacted by the diet of the mother, and therefore, except for calcium, by the use of dietary supplements [12]. In the present study, the dietary intakes of the mother were not recorded, and no recent survey on either dietary intakes or mineral status in Swiss pregnant or lactating women was retrieved for these three elements. Nevertheless, data on iodine intake in the general Swiss population was classified as sufficient by the Iodine Global Network (http://www.ign.org/scorecard.htm). In our study, this is reflected by an iodine median concentration in milk above the published median value of 62 μg/L [24]. A similar observation is made for calcium, despite the results of a Swiss cross-sectional study showing that in 28% of subjects following an omnivore diet, calcium intake does not reach the corresponding nutritional recommendation [33]. No recent data on the prevalence of intake inadequacy or deficiency was retrieved for selenium, but the rather low levels in milk observed in our study may indicate a need for a higher selenium intake in Swiss lactating women, as underlined in a review published earlier for the Swiss population [34]. This may help to increase the selenium milk content. In any case, even if dietary intakes of the mother do not influence their milk mineral concentration, they should give particular attention to the mineral density of their diet to match their own increased requirements if adaptation of the mechanism of absorption is not enough to avoid putting themselves at risk of mineral deficiencies.

The comparison of concentration at the same postpartum age shows no statistically significant difference between preterm HM and term HM for six minerals, i.e., potassium, zinc, iodine, selenium, iron, and calcium. These data are in agreement with previous published results for potassium [17,18,28,35,36], zinc [22,37,38], iodine [24,39], and selenium [26,40]. Few papers have reported higher levels of iron in preterm HM during the early lactation period [28,41,42], but some others [43,44] and a more recent one [45], failed to observe any differences. In HM, 2% to 9% of iron is linked to lactoferrin [45] and our iron content results correlate with the results of lactoferrin analyzed from the same set of samples [13]. Only minor and scattered differences between preterm HM and term HM have previously been reported for calcium. A recent systematic review and meta-analysis performed with 11 studies showed no differences from day 1 to week 12 postpartum, except at week 7 to 9, where a statistically significant difference of 15% was reported (preterm HM > term HM) [46]. Calcium along with phosphorus are the two main minerals required for the multifaceted mechanisms of bone metabolism [47]. Therefore, the meta-analysis was also performed for phosphorus from eight independent studies, and showed that the phosphorus concentration in preterm HM was not statistically different than in term HM, except between week 3 and 4 and week 5 to 6, with reported differences of 14% and 16%, respectively (preterm HM < term HM) [46]. In our study, a significant difference was only found at week 1 for this mineral in the opposite direction (preterm HM > term HM). We also found a higher concentration in preterm HM than in term HM for magnesium and only at three time points during the transitional phase (i.e., week 1 to 4). These differences were below 20%. In the literature, only one study out of 16 reported a similar difference [21,48]. In addition, we found lower concentrations of copper and sodium in preterm HM than in term HM, but only at postpartum week 2. Like for iron, inconsistent results were retrieved for copper in the literature [23]. Preterm HM copper concentrations were reported to be significantly lower [42,44], higher [41], or no different from the concentrations of term HM [44,49,50]. Except for one study [17], the sodium concentration in preterm HM is consistently reported as higher in preterm HM than in term HM, at the very early stage of lactation [18,51]. Our result goes in an opposite direction, but only at week 2. These discrepancies are most probably due to the difference in the timing of sample collection and comparison that, in most studies, started at day 1 postpartum versus day 7 in the present report. Other analytical reasons may be the differences in the number of samples, the collection method (e.g., foremilk vs. hindmilk as compared to full-breast expression in our study), and the storage procedures [45]. Beside this reading, although it is well-established that the levels of minerals in preterm HM are not sufficient to meet the needs of the preterm infant, it is not unusual to read that when higher concentrations are found in preterm HM than in term HM, it could be due to a physiological adaption to meet the increased requirement of the preterm infants [28,41,42,51]. This explanation goes along with the hypothesis of a reduced blood flow to the mammary gland and thus milk volume, and the immaturity of the gland (i.e., incomplete differentiation of epithelial cells, and the absence of tight junctions between the cells) [52]. However, some authors reported no difference in milk volumes produced by both preterm and term mothers, stressing that understanding of the physiological processes responsible for the micronutrient composition in human milk still requires investigation [17]. Some authors also report that it is likely that these discrepancies are due to differences in hormonal balance and metabolic regulation due to the shorter gestational period [17,53,54].

Overall, in agreement with previous reports, the present results show parallel compositional changes in the mineral content of milk from mothers of preterm and term infants throughout the 8 postpartum weeks of common sample collection. While breastfeeding is the gold standard for term and preterm feeding, for the latter, it is prescribed that human milk should be fortified with nutrients in short supply [7,8,9]. Despite the punctual differences described above, when comparing the median of values for each individual element to the recommendations by the ESPGHAN [9], it appeared that, except for iodine and potassium, none of the concentrations reached the minimum recommended level [9] (results presented in Supplementary Figure S1). This emphasizes the need for fortifying with only those minerals whose content in HM is lower than the recommended levels, in order to avoid overloads of those minerals that are present in HM at sufficient levels.

### 4.2. Longitudinal Changes of Mineral Concentration in Term and Preterm Human Milk at an Equivalent Postmenstrual Age

Fortification of human milk is recommended up to preterm infant discharge from the neonatal care unit, to address the mismatch between its content and the nutritional recommendations for optimal growth and development. However, there is a lack of consensus about the need to fortify human milk after discharge (usually occurring at around term corrected age) [10]. Considering that the references for an optimal growth of preterm infants after they reach the term corrected age are calibrated against the standards of infants born at term, we assumed that the preterm infant nutritional requirements during this period would be at least the same as those of their term counterparts. Therefore, the mineral contents of preterm HM were compared to those of term HM at the same postmenstrual ages (i.e., from postmenstrual weeks 39 to 48). This comparison showed highly significant differences for zinc, copper, and selenium, with a lower concentration in preterm HM than in term HM. Zinc and copper contribute to many cellular and molecular processes and therefore are essential for growth, the immune response, and cognitive function [4]. The serum zinc concentration in term and preterm infants is reported to be high at birth and then to progressively decrease, which was correlated with normal growth [55]. Selenium is an important factor for optimal function of the antioxidant systems and its serum concentration has been showed to increase in healthy term breastfed infants after birth [56]. Therefore, the observed differences of concentrations of these three trace elements in preterm HM and term HM at the same postmenstrual age may be physiologically relevant and require further investigation.

### 4.3. Strengths and Limitations of the Study

This study has some limitations. First, the samples were collected at only one time of the day. As the fluctuation of mineral concentration in milk has been previously observed over the day [29], one may argue that the data are not representative of the whole day concentration. Nevertheless, the sampling time was standardized and it allowed us to perform a comparison of the HM of the two infant populations at a postpartum and postmenstrual age. Finally, the number of subjects was limited in both groups, reducing the power of the study and limiting the number of association analyses.

## 5. Conclusions

This study represents the first set of simultaneous analyses of ten minerals in human term and preterm milk comprehensively covering the period of 2 months postpartum. It confirms previous observations of concentrations and temporal changes, as well as the global similarity in the composition of term and preterm milk when compared at the same lactation stage. Interestingly, zinc, copper, and selenium contents were found to be consistently lower in preterm milk than in term milk when compared at an equivalent infant developmental stage. The relevance of the observed differences to the health, growth, and development of preterm infants, as well as the impact on fortification practices, remain to be further investigated.

## Figures and Tables

**Table 1:** General characteristics of the two groups of mothers and infants.

Study Population	Preterm	Term	*p*-Value *
**Mothers (drop outs)**	N = 27 (2)	N = 34 (2)	
**Age (years)**	32.4 ± 5.6	31.2 ± 4.2	0.3173
**BMI before pregnancy (Kg/m^2^)**	22.8 ± 3.3	23.2 ± 4.9	0.6990
**BMI at child birth (Kg/m^2^)**	25.8 ± 3.7	26.9 ± 4.7	0.3141
**Delivery type: caesarean (%)**	63	23.5	0.0019
**Infants**	N = 33	N = 34	
**Gestational age at birth (weeks)**	30.8 ± 1.4	39.5 ± 1.0	<0.0001
**Males (%)**	54.5	52.9	0.8952
**Twins (%)**	36.4	0.0	0.0001
**Height (cm)**	40.4 ± 3.2	49.4 ± 1.7	<0.0001
**Weight (g)**	1421.4 ± 372.8	3277.6 ± 353.6	<0.0001
**Head circumference (cm)**	27.8 ± 2.1	34.4 ± 1.5	<0.0001

* *t*-test and Fisher test of proportions were used for the comparison of continuous and discrete variables, respectively.

**Table 2:** Summary of mineral concentrations (i.e., mean ± SD and median (min-max)) in preterm (from sample collection from postpartum week 1 to 16) and term human milk (as collected over 8 weeks postpartum). Retrieved published ranges of mineral concentration in human milk * are also provided for comparison.

Minerals	Statistic	Preterm Milk	Term Milk	Literature Range
(mg/L)				
Potassium	Mean	578 ± 107	575 ± 92	515^$^ [17], 688^$^ [18]
	Median	569 (209–907)	562 (308–908)	-
Calcium	Mean	281 ± 51	286 ± 47	-
	Median	282 (145–459)	287 (136–433)	252 (84–462)^#^ [19]
Sodium	Mean	205 ± 177	235 ± 237	135–371^#^ [20]
	Median	160 (54–1577)	170 (84–1969)	-
Phosphorus	Mean	145 ± 32	148 ± 30	-
	Median	145 (48–225)	146 (45–257)	143 (17–278)^#^ [19]
Magnesium	Mean	37 ± 9	32 ± 7	-
	Median	36 (12–70)	33 (17–53)	31 (15–64)^#^ [21]
Zinc	Mean	2.4 ± 1.7	3.2 ± 1.9	2.2–2.5; 2.9–3.9; 1.7–5.3^#^ [22]
	Median	2.1 (0.2–15.5)	2.7 (0.4–11.9)	-
Iron	Mean	0.36 ± 0.23	0.44 ± 0.26	-
	Median	0.32 (0.13–2.78)	0.36 (0.13–1.8)	0.47 (0.04–1.92)^#^ [23]
Copper	Mean	0.36 ± 0.16	0.44 ± 0.15	-
	Median	0.35 (0.06–1.1)	0.42 (0.13–1.0)	0.33 (0.03–2.19)^#^ [23]
**(µg/L)**				
Iodine	Mean	92 ± 67	87 ± 41	15–150^#^ [20]
	Median	76 (2–422)	76 (18–228)	62 (5.4–2170)^#^ [24]
Selenium	Mean	14.3 ± 4.7	15.0 ± 4.2	11^$^ [25]–28^$^ [26]
	Median	13 (4–45)	14 (6–34)	-

* Published median (min-max) value where preferred for reporting. ^#^ indicates that values were calculated from term human milk (HM); ^$^ corresponds to data calculated from preterm HM.

## Supplementary Materials

The following are available online at https://www.mdpi.com/2072-6643/11/8/1855/s1: Table S1: Concentration of human milk minerals and trace elements in term or preterm milk at different weeks postpartum; Table S2: Concentration of human milk minerals and trace elements in term or preterm milk at equivalent postmenstrual ages; Figure S1: Longitudinal changes of mineral concentration (means) in term (dark grey) and preterm (light grey) human milk (HM) at an equivalent postmenstrual age, with dotted lines representing the range of recommended intakes (converted in mg/L, using measured energy density of preterm HM), as provided by ESPGHAN [3].
