# Supplementary material for: Longitudinal Changes of Mineral Concentrations in Preterm and Term Human Milk from Lactating Swiss Women
Source: Nutrients. 2019 Aug 9;11(8):1855. doi: 10.3390/nu11081855 (PMC6723843; doi:10.3390/nu11081855)
Supplement: Supplementary file 1 [file nutrients-11-01855-s001.zip › Supplementary/Supplementary_Figure_1.pptx]

## Slide 1
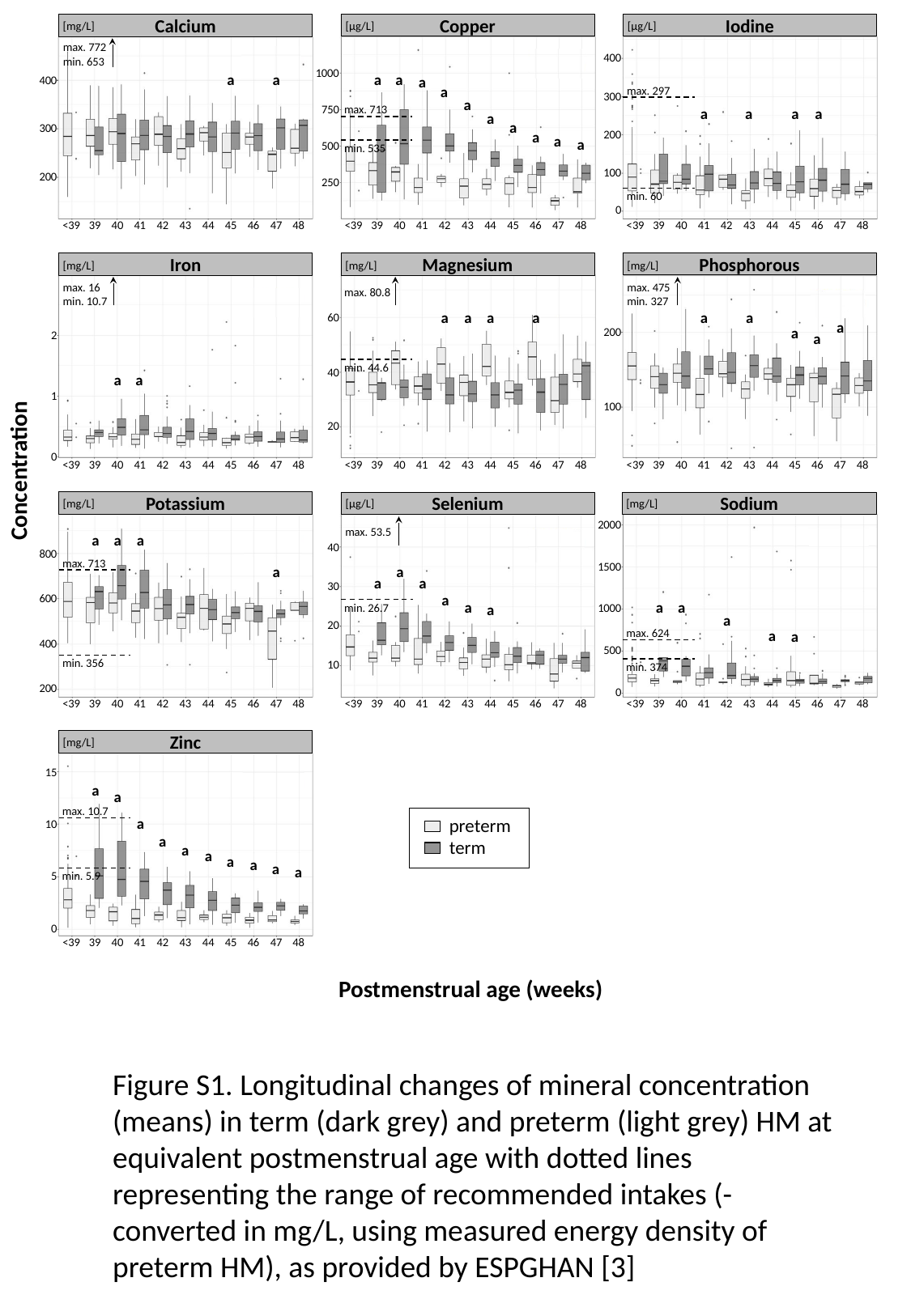

Copper
Iodine
Calcium
[mg/L]
[µg/L]
[µg/L]
max. 772
min. 653
400
1000
a
a
a
a
a
400
a
max. 297
300
a
750
max. 713
a
a
a
a
a
a
300
200
a
a
a
500
min. 535
100
200
250
min. 60
0
<39
39
40
41
42
43
44
45
46
47
48
<39
39
40
41
42
43
44
45
46
47
48
<39
39
40
41
42
43
44
45
46
47
48
Phosphorous
Iron
Magnesium
[mg/L]
[mg/L]
[mg/L]
max. 16
min. 10.7
max. 475
min. 327
max. 80.8
a
a
a
a
a
a
60
a
a
200
2
a
min. 44.6
40
a
a
1
100
20
0
Concentration
<39
39
40
41
42
43
44
45
46
47
48
<39
39
40
41
42
43
44
45
46
47
48
<39
39
40
41
42
43
44
45
46
47
48
Potassium
Selenium
Sodium
[mg/L]
[µg/L]
[mg/L]
2000
max. 53.5
a
a
a
40
800
max. 713
1500
a
a
a
a
30
a
600
a
a
a
min. 26.7
a
1000
a
20
max. 624
a
a
400
500
min. 356
10
min. 374
200
0
<39
39
40
41
42
43
44
45
46
47
48
<39
39
40
41
42
43
44
45
46
47
48
<39
39
40
41
42
43
44
45
46
47
48
Zinc
[mg/L]
15
a
a
max. 10.7
preterm
term
a
10
a
a
a
a
a
a
a
min. 5.9
5
0
<39
39
40
41
42
43
44
45
46
47
48
Postmenstrual age (weeks)
Figure S1. Longitudinal changes of mineral concentration (means) in term (dark grey) and preterm (light grey) HM at equivalent postmenstrual age with dotted lines representing the range of recommended intakes (-converted in mg/L, using measured energy density of preterm HM), as provided by ESPGHAN [3]
